# Pyrolysis-GC/MS differentiates polyesters and detects additives for improved monitoring of textile labeling accuracy and plastic pollution

**DOI:** 10.1007/s00216-025-05851-x

**Published:** 2025-04-01

**Authors:** Josh Forakis, Jennifer Lynch

**Affiliations:** 1 Center for Marine Debris Research, Hawaii Pacific University, 41-202 Kalanianaole Hwy #9, Waimanalo, HI 96744 USA; 2 National Institute of Standards and Technology, 41-202 Kalanianaole Hwy #9, Waimanalo, HI 96744 USA

**Keywords:** Microfibers, Polyesters, Py-GC/MS, Polymer identification

## Abstract

Polyesters comprise the greatest proportion of textile fibers and are found in various everyday goods; hence, polyester fibers are a significant source of microplastic pollution and textile waste. The specific chemical composition of commercial polyester fibers is often proprietary and mostly assumed to be poly(ethylene terephthalate) (PET). Polyester is a class of polymers that include poly(butylene terephthalate) (PBT), poly(cyclohexylenedimethylene terephthalate) (PCT), and poly(ethylene naphthalate) (PEN), as well as biodegradable polymers. Our study aims to clarify whether household polyester products are primarily PET, are labeled accurately, or contain phthalate additives by applying double-shot pyrolysis–gas chromatography/mass spectroscopy (Py-GC/MS). We analyzed four scientific-grade polyester reference standards, 52 manufacturer-grade polyester fibers or pellets, and 229 samples from 193 consumer polyester products. From the pyrograms, samples were predominantly identified as PET (87.4%, 95% CI [93.5–81.3%]), but five samples were identified as a different polyester, nine as non-polyester polymers, and 23 as a blend of PET with another polymer. From the thermal desorption chromatograms, diethyl phthalate was the most frequently detected phthalate, found in 23.3% (95% CI [17.3–29.3%]) of the consumer products, including children’s toys. Double-shot py-GC/MS advantageously results in these empirical data that (1) counter the assumption that products labeled polyester are always PET, (2) emphasize the importance of creating spectral libraries with well-characterized materials for accurate polymer identification of unknown plastic particles, and (3) demonstrate that phthalates are common additives in household products.

**Graphical Abstract:**

Pyrolysis-GC/MS clarifies commercial polyester compositions and phthalate content, informing microplastic pollution monitoring and product labeling policies for improved environmental health.

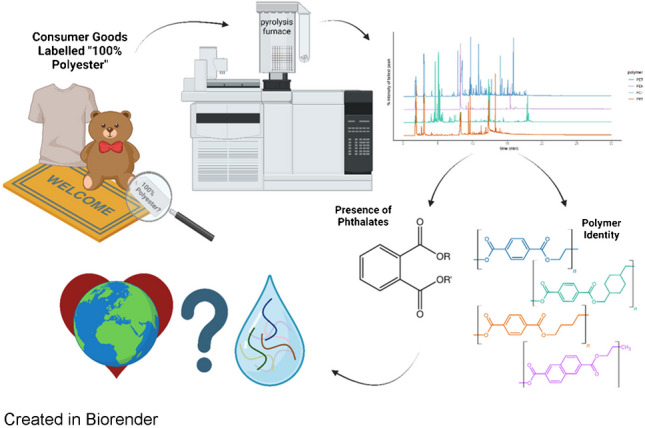

**Supplementary Information:**

The online version contains supplementary material available at 10.1007/s00216-025-05851-x.

## Introduction

Microfibers, defined by pollution researchers as natural or synthetic fibrous materials of a thread-like structure with a diameter of less than 10 μm, a length between 1 μm and 5 mm, and a length-to-width ratio greater than 100, are becoming the most abundant form of microparticle pollution identified in the natural environment [1, 2]. Whole fabrics are also found as marine debris or plastic pollution, impacting coral reefs and other ecosystems. Many common textile materials shed microfibers during production, use, and end-of-life [3, 4]. Natural fibers, like cellulose and silk, are biodegradable, so they are less of an environmental concern [5]. Still, synthetic textiles, commonly made of polyester, acrylic, nylon, or polyolefins, are designed to last, and thus, those products or microfibers that leach from them persist in the environment. Microfibers have been identified in all environmental matrices, including the atmosphere, wastewater, sludge, and marine sediment [6–9]. In addition, 0.28 million tons of microfibers is estimated to be released into aquatic environments yearly [10]. Aquatic organisms can mistake microfibers for food, which can negatively impact ecological and human health by leaching small-molecule contaminants, diluting nutrients, and bioaccumulating or biomagnifying plastic particles in biota and up the food chain [11, 12].

**Fig. 1:**
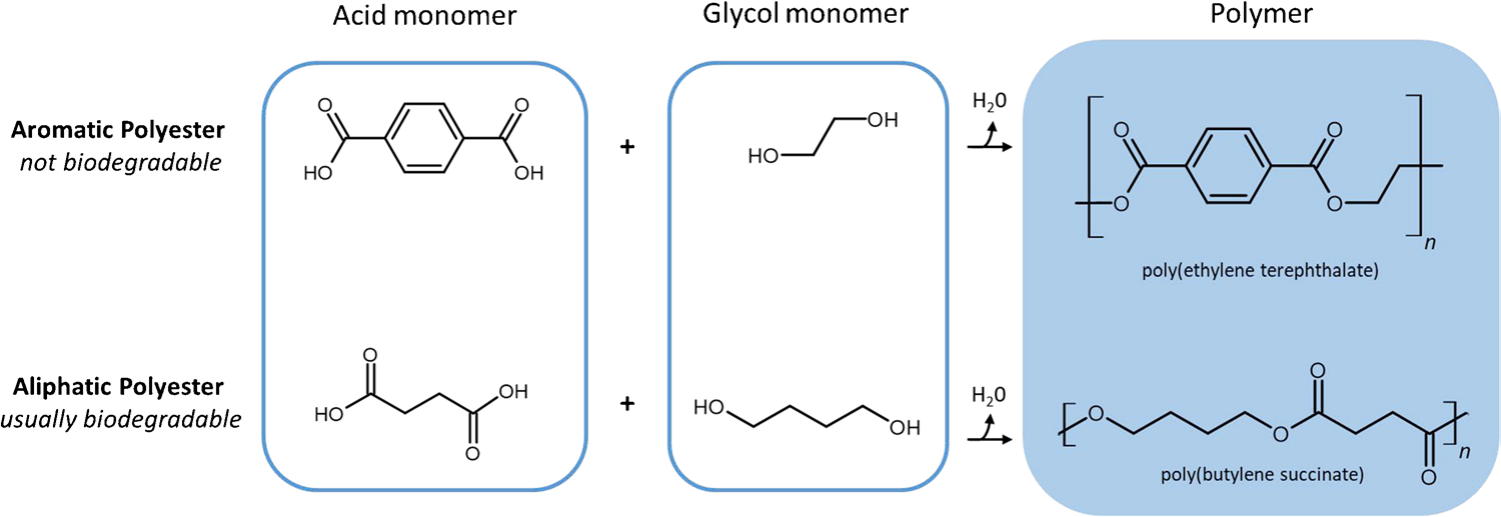
Two types of polyester compounds. Polyesters often comprise two monomers: a difunctional carboxylic acid (acid monomer) and a difunctional alcohol (glycol monomer). These monomers can undergo a condensation polymerization reaction to create polyester compounds, releasing water as a byproduct. The acid component contains an aromatic functional group for aromatic polyesters, and the resulting polymer is not biodegradable. Aliphatic polyesters are typically biodegradable and do not contain aromatic functional groups in their acid monomer

The production rate of synthetic fiber textiles exceeds that of natural fibers and continues to grow. Polyester accounted for 54% of all globally produced fibers in 2022 (not just synthetic), and polyester textiles are used in various everyday products, including clothing, carpets, curtains, and toys [13]. The traditional definition of a polyester is any polymer with an ester group in the repeating units of the main polymer chain [14]. Polyesters can be further categorized into those containing a difunctional ester of an aromatic substituted carboxylic acid (usually terephthalic acid) and those with an ester group but no substituted aromatic carboxylic acid monomer. The category with a substituted aromatic carboxylic acid monomer is the most common for commercial textile fiber applications. These polyesters include poly(ethylene terephthalate) (PET plastic resin code #1) and poly(butylene terephthalate) (PBT), which do not biodegrade in the environment [15]. Polyesters with an aliphatic carboxylic acid monomer, or aliphatic polyesters, are primarily hydrophobic, biodegradable or compostable polymers because their ester groups are readily hydrolyzed by microbes in the decomposition process [16, 17]. Figure 1 displays an overview of the two categories of polyesters. Only aromatic polyesters are considered for this study, as these are the most relevant regarding microfiber pollution.

Aromatic polyesters are copolymers containing two monomer types: a substituted aromatic carboxylic acid and a glycol, joined via condensation polymerization [14]. Different types of aromatic polyesters were designed by making minor structural alterations to the carboxylic acid or, more commonly, the glycol monomer. Table 1 gives an overview of four common aromatic polyesters: PET, PBT, poly(1–4 cyclohexylenedimethylene terephthalate) (PCT), and poly(ethylene naphthalate) (PEN). PET, PCT, and PBT contain the same carboxylic acid monomer but contain different glycol monomers: ethylene glycol, cyclohexanedimethanol, and 1,4-butanediol, respectively. PEN is made with ethylene glycol but has an altered carboxylic acid monomer, dimethyl-2,6-naphthalenedicarboxylate. Due to the chemical differences in their polymer backbone, different polyesters offer a range of mechanical and chemical properties, including heat and chemical resistance and the ability to be molded into different products [18, 19].

**Table 1:**
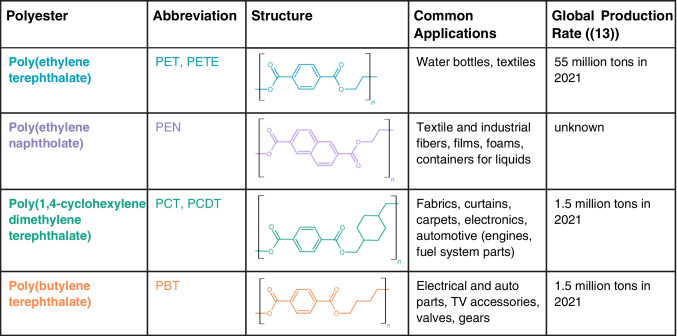
Structure, common applications, and production rates of common aromatic polyesters

Various analytical techniques have been employed to identify and quantify environmental microfibers [20]. Spectroscopic techniques are the most common, but they are limited in their ability to differentiate polymers with the same functional groups [21]. This means that the spectra of the different polymers in the same group, such as polyesters and polyamides (nylon), look similar and may only differ by minor, easy-to-miss spectral features [22, 23]. Furthermore, a low-quality spectrum of one polymer type might cause it to register as a different polymer according to a spectral correlation search. In addition, plastic pollution laboratories, like ours, create spectral libraries using consumer goods labeled with their polymer composition. Since polyester is a common label on textile goods, spectra of these materials are entered into libraries under this umbrella term. Polyester can also be found in commercial spectral libraries without explaining the specific polymer used in that entry. Plastic pollution pieces often match these polyester spectra, which does not provide complete chemical information about the pollution.

Thermal decomposition methods such as Py-GC/MS and thermal extraction and desorption GC/MS (TED-GC/MS) offer advantages for plastic pollution analysis [20, 24–26]. Breaking the bulk polymer chain into characteristic fragments provides more detailed information on the sample’s chemical structure, including specific monomer subunits. Unlike spectroscopic methods, they destroy the sample, require a small amount of material, and provide polymer mass rather than particle count. TED-GC/MS is a two-step technique combining thermo-gravimetric analysis and solid-phase absorption with thermal desorption GC/MS. This method allows for sample sizes up to and above 100 mg, but is less straightforward than Py-GC/MS, with TED-GC/MS requiring solid-phase extraction before desorbing and analyzing fragments. Py-GC/MS directly pyrolyzes the sample in a furnace before passing the compounds through the GC/MS system, but is limited to samples less than 0.5 mg. Py-GC/MS is a useful technique when the sample is relatively pure and requires little preparation. Py-GC/MS can differentiate polymers with the same functional groups, including polyesters, and it has been employed in microfiber research to characterize textile fibers and quantify fiber release from laundering polyester clothes [27, 28].

The precise bulk polymer composition of polyester products is often undisclosed, most frequently labeled “100% polyester fibers” or something similar. From the perspective of the plastic pollution research community, this can cause challenges when building or using infrared or Raman spectral libraries to quantify and characterize environmental microfibers [29, 30]. Researchers rely on spectral libraries to assign a polymer identity to unknown plastic samples, often making their spectral libraries from readily available consumer goods and naming that spectra the polymer or material type printed on the label. Reference spectra from polyester-labeled materials are ambiguous to the actual polymer. For example, several microplastic studies have reported detecting both polyester and PET [31–34], which implies that they are using a library that contains a spectrum from an ambiguous polyester material and cannot identify all of the polyester particles to a specific polymer. Accurate environmental monitoring depends on quality reference libraries made with the most fully characterized materials.

Thus, this study was designed to test a common assumption that most polyester products, and therefore pollution, consist of PET [3, 35, 36]. While PET is the most commonly produced polyester, assuming all polyesters to be PET overlooks the diversity of polyester polymers and may affect accuracy of plastic pollution studies. This study employed Py-GC/MS to identify the precise bulk polymer composition of four scientific standard polyesters, 52 polyester fibers representative of a wide array of commercially available fibers from numerous producers across the globe (Forensic Fiber Reference Collection by Microtrace LLC), and 229 samples from consumer goods labeled “100% polyester.” While we predicted most polyesters would be identified as PET, no previous study has applied the advantages of Py-GC/MS to empirically identify the polymer of scientific-grade, manufacturer, and consumer-good polyester fibers. These data are a necessary contribution towards harmonizing plastic pollution analysis and improving the accuracy of microfiber quantification. Furthermore, the thermal desorption chromatograms produced by double-shot Py-GC/MS offered an opportunity to simultaneously inspect the samples for the presence of some plastic additives that are commonly added at multiple steps of plastic product manufacturing [37].

## Methods

### Sample acquisition

All materials acquired and analyzed in this study are listed in Online Resource 1. Scientific polyester standards of poly(ethylene terephthalate) (PET) and poly(butylene terephthalate) (PBT) were purchased from Scientific Polymer Products Inc. and used as reference standards. The purchase of a scientific source of polycyclohexylene dimethylene terephthalate (PCT) was intended from Nanochemazone, but this standard was later identified as PET, a different polyester.

Manufacturer-grade polyesters were obtained from the Forensic Fiber Reference Collection by Microtrace LLC (Fiber Library). The Fiber Library contained 52 fibers labeled “polyester” from 10 or more companies, representing a similar proportion of polyester types produced globally (roughly 95% PET, 5% other polyesters). The PCT fiber from Bayer (Fiber Library #135) and the PEN fiber from Allied Signal Fibers (Fiber Library #133) were used in this study as reference standards for these additional polyester polymers. When a specific product usage (e.g., carpet) was specified in the manufacturer description of fibers in this library, this was entered into the consumer product category column of the spreadsheet. Two carpet fibers, two clothes fibers, and four miscellaneous fibers from the Fiber Library were included in the consumer product categories outlined below.

Consumer products, or components of these products, labeled as “100% polyester,” were obtained from stores or our homes as samples. Of the 237 consumer samples analyzed for this study, including Fiber Library fibers with a specified usage, 12 were bags, 25 were bedding (pillows, pillow covers, blankets, sheets, etc.), 48 were carpets, 49 were clothes, 24 were curtains, 67 were toys, and 12 were categorized as “miscellaneous,” which included a washcloth, towel, Christmas stocking, fabric not sewed into a product, fishing nets, camping gear, and beach chair fabric. Before collecting fiber samples, we decided whether to sample from one of three possible sample components: the exterior (outward-facing material of the product), the interior (inward-facing material of the product, liner fabric), or the stuffing (fibers specifically used to fill the inside of a product like pillows or stuffed toys). Around 1 mg to 3 mg of the material was cut and stored in paper envelopes or LDPE plastic bags until sample preparation. The most abundant category of sample component was the exterior (*n* = 165), followed by stuffing (*n* = 41) and interior (*n* = 24) samples. The Fiber Library fibers (*n* = 52) and scientific standards (*n* = 4) were not assigned a sample component.

### Sample preparation

All samples were prepared on a marble slab in a clean, controlled laboratory environment. The marble slab and all materials used were rinsed with a 70% isopropanol solution before and between samples. Between 0.1 mg and 0.5 mg of fibers from each sample were cut using a metal razor blade and transferred with metal forceps into a tared stainless steel pyrolysis cup (Frontier Labs P/N: PY1-EC80F). Sample mass was recorded on a microanalytical balance (Mettler-Toledo XPR6U, resolution 0.1 μg). A glass fiber filter (Osmonics Inc.; ⌀ = 25 mm; pore size = 0.1 µm; P/N G40WP02505) was baked at 450 °C for 4 h, then cut into fourths using a metal razor blade. One-fourth of the filter was folded in half, rolled up using a pair of metal forceps, and placed on top of the fibers in the pyrolysis cup to hold them in place. Packed pyrolysis cups were stored in a covered, aluminum foil-lined tray until analysis.

### Instrumental methods

Pyrolysis analysis of the samples was performed on an Agilent gas chromatograph (GC, Agilent, 8890) coupled with a mass spectrometer (MS, Agilent, 5977B) and a Frontier Multi-shot pyrolyzer (EGA/PY-3030D) with an auto-shot sampler (AS-1020E). All samples were analyzed using a two-step method, including thermal desorption (TD) and pyrolysis. The TD range removes low molecular weight molecules in the polymer matrix but not bound to the bulk polymer, such as additives and leftover monomer molecules [25]. The pyrolysis temperature breaks apart the bulk polymer compound into pyrolyzates indicative of the parent compound. Online Resource 2, Table S1 includes the precise parameters of the double-shot method used on all samples. To complement the analysis, the standard polyesters were analyzed on via attenuated total reflectance Fourier-transform infrared spectrometer (ATR-FTIR) (Thermo Fisher Nicolet Summit X).

### Identification of bulk polymer

Agilent Masshunter software was used to visualize pyrograms (Qualitative Analysis) and collect and analyze chromatographic peaks (Quantitative Analysis). Bulk polymer characterization was performed utilizing the Pyrolysis-GC/MS Data Book of Synthetic Polymers, the National Institutes of Standards and Technology MS Program, and the Frontier F-search software [38–40]. First, the shared and unique pyrolyzates of different polyesters were identified. Next, the pyrograms of the four reference standards of PET, PCT, PBT, and PEN were opened in Qualitative analysis, and the total ion chromatogram (TIC) was visualized, showing the amount of all compounds eluted at each retention time. Extracted ion chromatograms (EICs) were created to identify expected retention times by extracting mass ions relative to unique polyester pyrolyzates. EICs show the abundance of one target ion that eluted at a particular retention time. Mass spectra were then generated from the extracted pyrolyzate peaks to identify appropriate target and qualifier ions. These peaks and their mass spectra confirmed the polymer identity shown on the reference standard labels compared to the Pyrolysis-GC/MS Data Book of Synthetic Polymers. Response ratios of the qualifier ions to the target ion were determined by visually inspecting the extracted mass spectra of the four reference standards. Pyrolyzates were confirmed by searching the generated MS data against the installed database. The retention times of linear n-alkanes 7–28 were determined from a sample of pure polyethylene (HPU CMDR Polykit 1.0), demonstrated as a reliable source for n-alkanes when using py-GC/MS [41]. A small amount (0.198 mg) was loaded into a stainless steel pyrolysis cup and analyzed under the same program and column described in the previous section. Kovat’s retention indices were then calculated from the retention times determined from standard materials and the retention times of linear n-alkanes [42]. Shared and unique polyester pyrolyzates, their retention index, and ion response ratios are shown in Online Resource 2, Table S2.

An automated method in the Quantitative Analysis software using parameters was created to extract peak areas and calculate response ratios of targeted polyester pyrolyzates from consumer goods and manufacturer-grade samples. A polyester pyrolyzate was considered present in the pyrogram if the target mass ion and qualifier ions were present at the same retention index with the same response ratio (20% uncertainty) as the corresponding pyrolyzate in the confirmed reference standard. When both criteria were satisfied for all pyrolyzates of a polymer, the bulk polymer identities were manually assigned to each sample. For example, a sample identified as PET must have all four unique PET pyrolyzates confirmed in its pyrogram and not contain unique pyrolyzates for other polymers. A polymer was considered a blend if all the unique pyrolyzates for two or more polymers were detected and confirmed in its pyrogram. That is, a PET/PCT blend must contain all four unique PET pyrolyzates and all three unique PCT pyrolyzates, and it must not contain unique pyrolyzates from other polyesters.

Samples that could not be assigned a polymer identity were analyzed again on Py-GC/MS. If the second pyrogram was still unidentifiable, it was searched using the Frontier F-search software. The top hits of the F-search were examined to provide a direction for manually identifying the unknown sample’s polymer. Then the pyrogram was opened in Qualitative Analysis, and the Py-GC/MS Data Book of Synthetic Polymers was used to identify mass ions to extract from the direction of the F-search results. Characteristic pyrolyzate identities were confirmed by running extracted mass spectra through the NIST Mass Spectral Search Program for the NIST/EPA/NIH EI and NIST Tandem Mass Spectral Library, Version 3.0 (NIST MS Program) until its identity was narrowed down to one bulk polymer or polymer blend.

### Identification of phthalate plastic additives

Information about plastic additives in polyester samples was obtained from the chromatogram of compounds that evolved in the TD range [25]. For this study, all samples were screened for phthalate compounds often added to polymers to increase plasticity [43]. NIST Standard Reference Material 3074: Phthalates in Methanol (NIST SRM 3074) was analyzed on Py-GC/MS to determine the expected retention index of several phthalate compounds, including di-*n*-butyl phthalate (DBP), benzyl butyl phthalate (BBP), *bis*(2-ethylhexyl)adipate (BEHA), *bis*(2-ethylhexyl)phthalate (BEHP), and di-*n*-octylphthalate (DOP). 80 μL of NIST SRM 3074 was delivered into a clean, stainless steel pyrolysis cup and set in a fume hood overnight to evaporate. The sample was then analyzed using the previously described thermal desorption program (100 °C to 200 °C by 20 °C/min). An additional standard of diethyl phthalate (DEP) (Accustandard; P/N APP-9–081) was purchased and then prepared and analyzed using the same method.

An automated method on Masshunter Qualitative Analysis was created by analyzing NIST SRM 3074 plus DEP, which was applied to the TD chromatogram of all samples after Py-GC/MS analysis (Table 2). The extracted mass spectra from each sample at retention indices established from the phthalate reference standards were examined. A phthalate was considered present if it was at the correct retention index and its mass spectra contained the target ions in the defined response ratios within a 20% uncertainty.

**Table 2:**
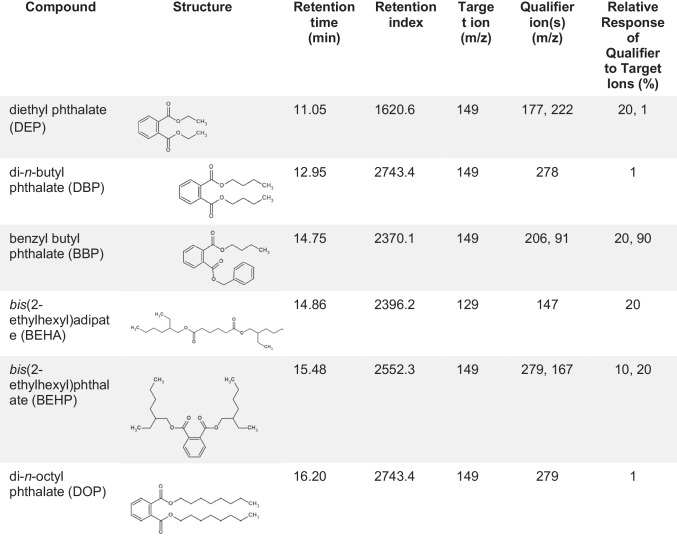
Structures, retention index, target, and qualifier ions of phthalates as determined by analyzing NIST SRM 3074 and DEP standard by pyrolysis-GC/MS

### Statistical methods

All statistical tests were performed in JMP (JMP Statistical Discovery, Cary, NC). Proportions are presented as percentages with 95% confidence intervals (CI). To compute these intervals, we applied the normal approximation with a finite population correction [44]. Specifically, for a given proportion, $p$, the 95% confidence interval was calculated as:

$$CI\;=\;p\;\pm\;z_{a/2}\sqrt{\left(\frac{p(1-p)}n\right)\;\left(\frac{N\;-\;n}{N\;-\;1}\right)}$$

where

- $X_{a/2}$ is the critical value from the standard normal distribution (1.96 for α = 0.05),
- $n$ is the sample size,
- $N$ is the population size (100,000), and
- $\frac{N-n}{N-1}$ is the finite population correction term.

Chi-squared $\left(X^2\right)$ tests for homogeneity were used to determine whether the proportions of polymer IDs differed among consumer product categories or sample components. Then, the proportions of polymer ID results were compared among pairs of consumer product categories or sample components using a $X^2$ test for independence. Finally, $X^2$ tests for independence were performed to determine whether the proportion of each polymer ID differed from that of the same polymer in all other consumer product categories or sample components combined. If the expected value for any group was below 5, Fisher’s exact test for independence was used instead. Differences in proportions between groups were considered statistically significant if *p* < 0.050.

## Results and discussion

### Differentiating aromatic polyesters with py-GC/MS and ATR-FTIR

**Fig. 2:**
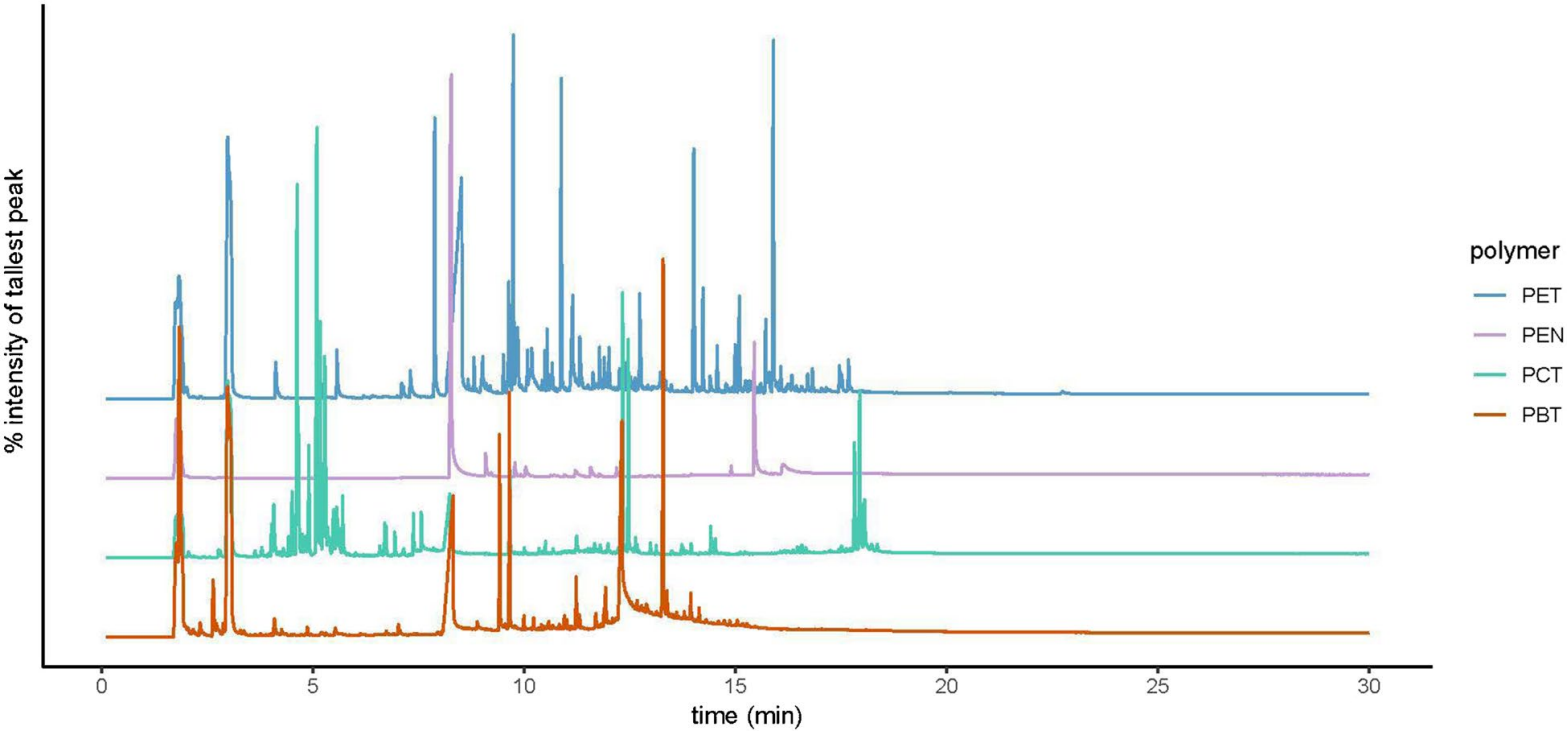
Total ion pyrograms of polyester standards were used to determine retention indices of pyrolyzates. Polyester standards were Scipoly poly(ethylene terephthalate) (PET), Fiber Library #133 Polyester Allied Signal Fibers (PEN), Fiber Library #135 Polyester Bayer (PCT), and Scipoly poly(butylene terephthalate) (PBT). All pyrograms are scaled to the tallest peak in each pyrogram

Most, but not all, scientific and manufacturer-grade standards were labeled accurately (Online Resource 1). The Nanochemazone PCT scientific standard was identified as PET by Py-GC/MS. This mislabeling could have caused persistent reoccurring inaccurate polymer identifications of unknowns if the standard had been added to in-house or commercial spectral libraries. To overcome our lack of a PCT scientific standard, we substituted a manufacturer-grade PCT standard from the Microtrace Fiber Library (Fiber Library #135) to explore pyrolyzates that permit differentiation among PCT, PET (SciPoly), PBT (SciPoly), and PEN (Fiber Library #133) (Fig. 2).

**Fig. 3:**
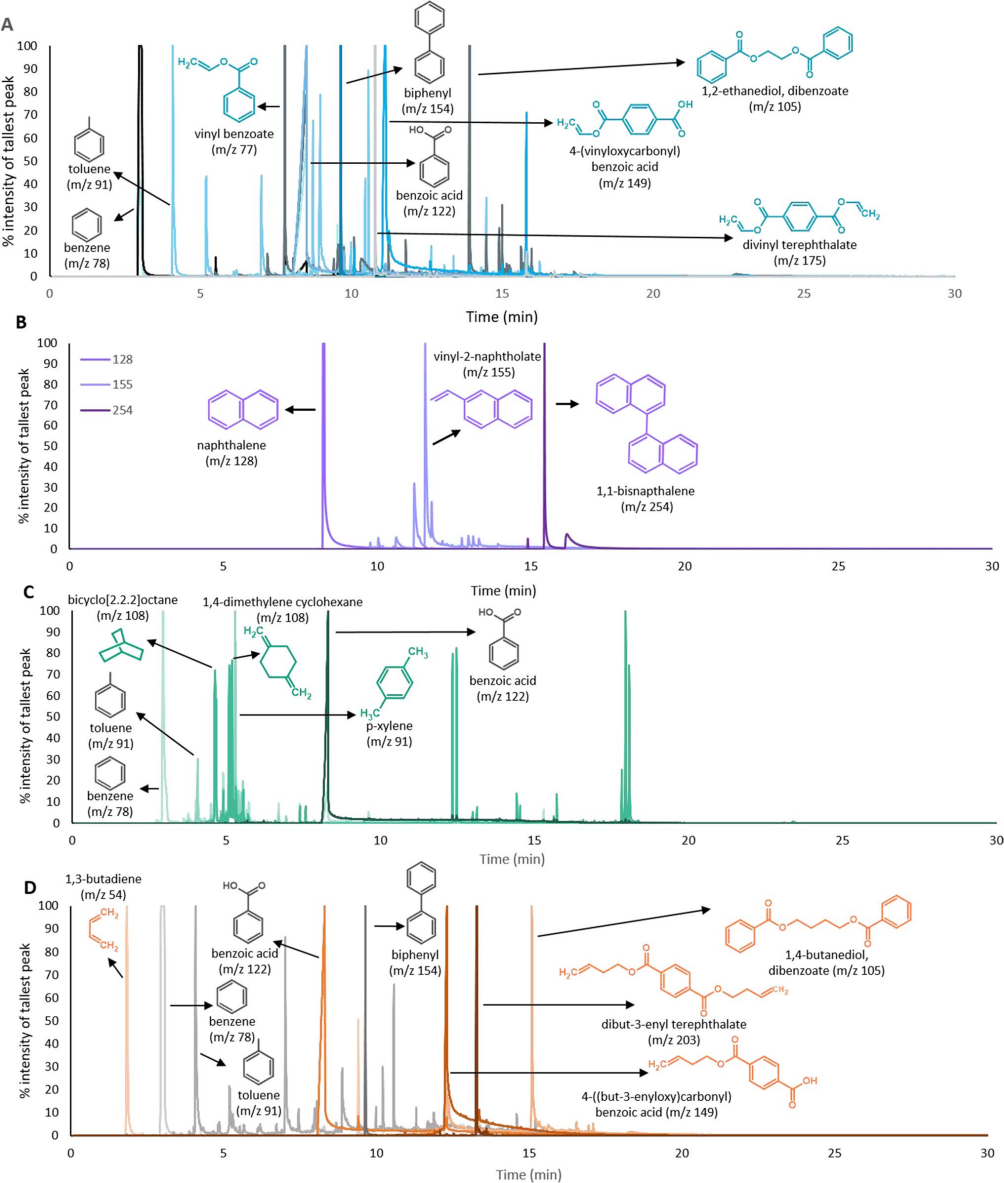
Extracted ion chromatograms and pyrolyzate structures of **A** PET, **B** PEN, **C** PCT, and **D** PBT reference standards. Pyrolyzate structures are shown with the mass charge ratio (m/z) extracted to identify that compound. Pyrolyzates unique to the polymer are in color, and shared pyrolyzates are shown in gray

A strong vinyl benzoate peak in the pyrogram at retention index (RI) = 1143.6 was reliable and unique to PET and served as a straightforward PET indicator. PCT shares several pyrolyzates with PET, including benzene, toluene, and benzoic acid. However, several unique pyrolyzates around an RI of 850 indicate the 1,4-cyclohexanedimenthanol monomer, including bicyclo[2.2.2]octane and 1,4-bis(methylene) cyclohexane. PBT shares several pyrolyzates with PET and PCT but has one unique low molecular weight pyrolyzate originating from the 1,4-butanediol monomer (RI = 610.8, 1,3-butadiene) and two others between RI = 1200–2500. Due to PEN’s unique acid component, all of its pyrolyzates are unique from other aromatic polyesters, with the most prominent being naphthalene (RI = 1203.0). Larger pyrolyzates at later retention indices are unique among polyester species as they typically combine the parent polyester’s acid and glycol monomer. This is also true for PCT, though not shown because PCT’s high molecular weight pyrolyzates have not been characterized [39]. Figure 3 shows the pyrolyzate structures and EICs of PET, PCT, PBT, and PEN standards.

The ATR-FTIR spectra of the four polyester standards demonstrate the general similarities (see Online Resource 2, Figure S1). These can be differentiated through close inspection of particular unique bands. PET has a unique band at 1042 cm^−1^. PBT has a unique band that overlaps with the others but peaks at 936 cm^−1^ and is missing the peaks unique to PET and PCT. PCT has prominent bands at 601 cm^−1^ and 2850 cm^−1^ and a broad band between 900 and 1000 cm^−1^ that peaks at 956 cm^−1^. PEN is the most different from the other three with unique bands at 1601 cm^−1^, 1339 cm^−1^, 1178 cm^−1^, and 918 cm^−1^. These differences among PET, PBT and PCT were also evident in spectra of these polymers in commercial libraries.

### Identifying polyester blends

Of this study’s 281 consumer and manufacturer-grade samples, 23 were identified as a blend of two or more polymers. Py-GC/MS is a quantitative technique, and the chromatogram’s peak area correlates to the amount of one pyrolyzate relative to the others coming off that sample [25]. As such, the peaks for one polymer’s pyrolyzates in the TIC had much greater intensity than the other in the blend. The polymer of a lesser amount in the blend could only be detected after examining EICs relevant to the less intense polymer in the blend. Online resource 2, Figure S2 shows the TIC and EICs of a PET/PCT blend (A: Fiber Library #147) and a PET/PCT/PBT blend (B: Fiber Library #185). The unique pyrolyzates for PCT (RI ~ 850) were somewhat visible in the TIC but were much less intense than the PET pyrolyzates (m/z = 105). When the mass ion for bicyclo[2.2.2]octane and 1,4-bis(methylene) cyclohexane was extracted (m/z = 108), the presence of PCT pyrolyzates was more evident.

### Fiber library

Of the 52 manufacturer polyester fibers from the Microtrace Fiber Library, 51 were identified as polyester or polyester blends (Online Resource 1). The remaining sample was poly(hexamethylene adipamide) or nylon-6,6 (PA66; Table 3). The Fiber Library fibers with a manufacturer description as an unspecified polyester were all PET or a PEST blend; thus, the manufacturer descriptions of Microtrace Fiber Library fibers are accurate, except for #149 Polyester Grilon SA, which was identified as PA66.

**Table 3:** Polymer ID of Fiber Library Polyesters according to Manufacturer Label and Py-GC/MS Results

Polymer on label	Count of samples in Microtrace Fiber Library	% (count) of samples correctly labeled as identified by Py-GC/MS	% (count of identified polymers) of samples incorrectly labeled as identified by Py-GC/MS
PET	29	96.6% (28)	3.4% (1 PA66)
PBT	2	100% (2)	0%
PCT	1	100% (1)	0%
PEN	1	100% (1)	0%
PEST	18	100% (12 PET, 4 PET/PCT, 2 PET/PCT/PBT)	0
PEST copolymer (Copolymer terephthalic acid + ethylene glycol + p-hydroxybenzoic acid)	1	100% (1 Copolymer terephthalic acid + ethylene glycol + p-hydroxybenzoic acid)	0
Total	52		

### Polymer identities of polyester fibers by consumer product category and sample component

**Fig. 4:**
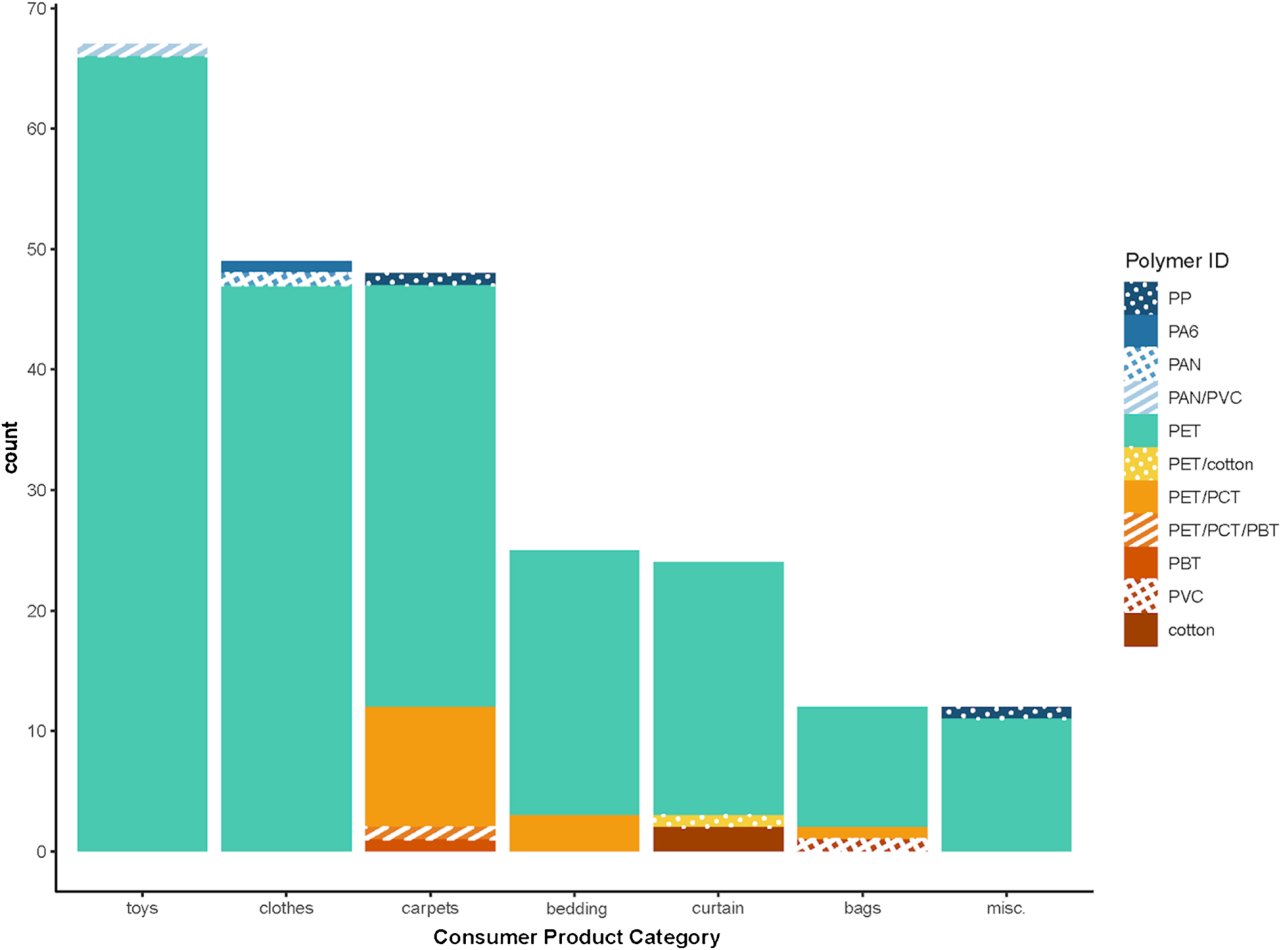
Polymer identification of consumer products labeled as polyester. Blends are described by listing all polymers identified in the pyrograms but do not provide the relative amounts of those polymers in the blend

For samples of consumer products made with polyester fibers, PET was, as expected, the most commonly identified polymer among all sample types (Fig. 4). For eight samples, F-searches were performed because they could not be initially identified (Online Resource 2, Table S3). Toys were 98.5% PET (95% CI shown in brackets throughout) [54.7% to 77.3%], with one sample identified as a blend of poly(acrylonitrile) and poly(vinyl chloride) (PAN/PVC: 1.5% [0% to 3.4%]). Clothes were 95.9% PET [90.4% to 100%], and the other two samples were PAN and poly(caprolactam) or nylon-6 (PA6). Carpets were identified as 72.9% PET [60.3% to 85.5%] and 20.8% PET/PCT [9.3% to 32.3%]. The remaining three carpet samples were identified as PBT (2.1% [0% to 6.2%]), PET/PCT/PBT (2.1% [0% to 6.2%]), and polypropylene (PP: 2.1% [0% to 6.2%]). Bedding samples were identified as 88% PET [75.3% to 100%] and 22% PET/PCT [5.8% to 38.2%]. Curtains were identified as 87.5% PET [74.3% to 100%] and 8.3% cotton [0% to 19.3%]. The remaining curtain sample was identified as a blend of PET and cotton. Finally, the miscellaneous samples were identified as 91.7% PET [76.1% to 100%], and the remaining was PP.

Polymer ID proportions were significantly different among consumer product categories, so further analysis was considered (see Online Resource 2, Table S4). Carpet fibers contained a greater proportion of PET/PCT blends than the other product categories, and their polymer composition was significantly different from clothes, curtains, and toys (see Online Resource 2, Table S5 and S6).

**Fig. 5:**
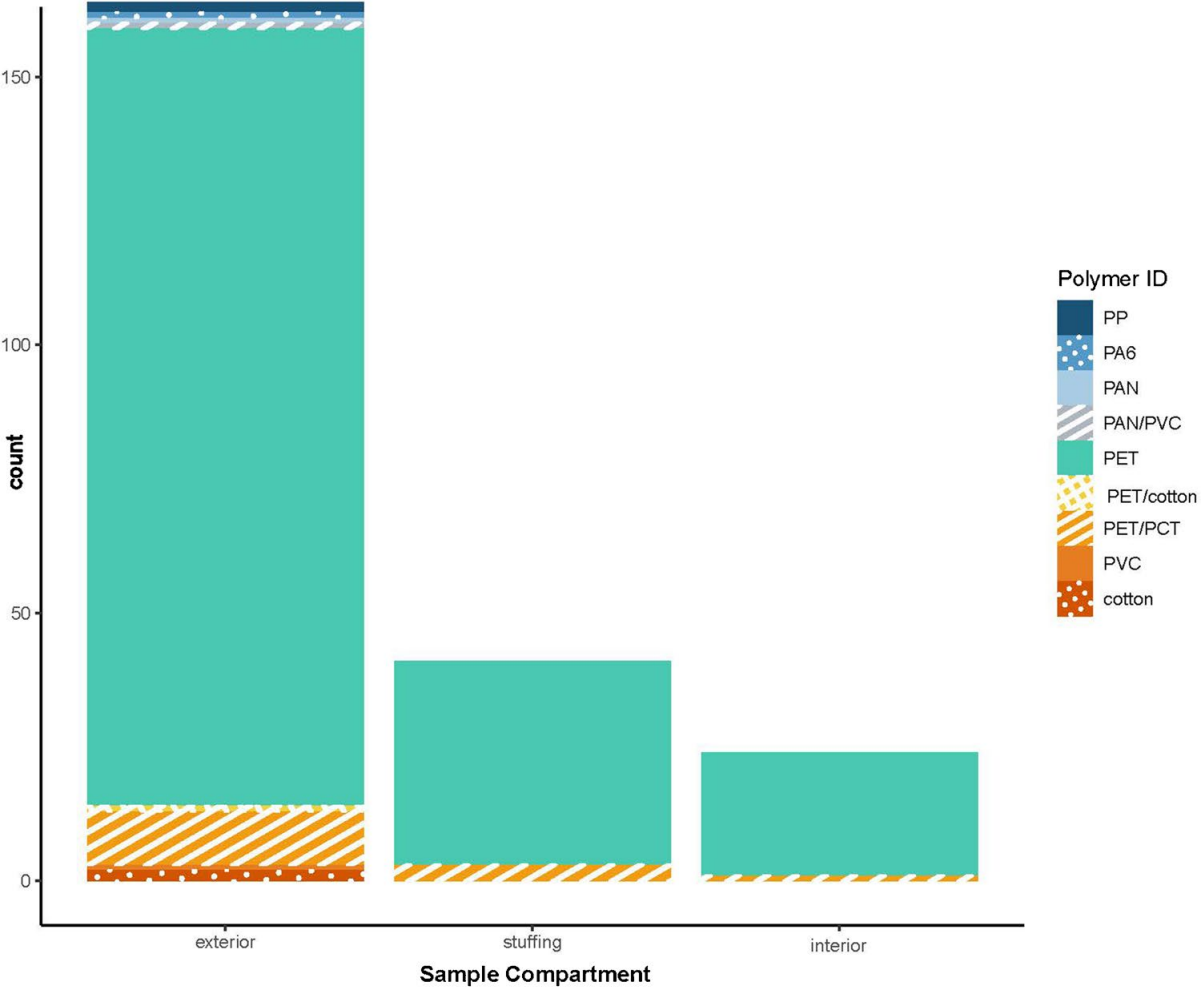
Polymer identification of components of consumer products labeled as polyester. All samples from consumer products were given one of three descriptions for sample component. Blends are described by listing all polymers identified in the pyrograms but do not provide the relative amounts of those polymers in the blend

The proportions of polymer IDs among sample components were not significantly different (see Online Resource 2, Table S7 and S8). Most samples were collected from the exterior because some products did not contain stuffing or an interior component (Fig. 5). In all but two of the 20 products that had paired sample components, the polymers were identical across the components and were identified as PET. The exceptions were a bed pillow stuffed with a PET/PCT blend fill but upholstered with PET fabric and a backpack constructed of seven components. Six components were PET, but the exterior thin film used for decoration and stiffness was PVC. All samples’ stuffing and interior components were identified as 92.6% [84.6% to 100%] and 95.8% [87.8% to 100%] PET, respectively. The remaining 7.3% [0% to 15.3%] and 4.2% [0% to 12.2%] blended PET and PCT.

The fibers sampled from the exterior component of products were 88.4% [83.5% to 93.3%] PET or 6.1% [2.4% to 9.8%] PET/PCT. Interestingly, the exterior was the only component where non-polyesters were identified (PA6, PP, PAN, PAN/PVC, PVC, and cotton). Also, the exterior was the only category that contained a polyester and non-polyester blend (PET/cotton from a curtain sample). These results confirm the assumption that most products labeled as polyester are PET, but it is important to acknowledge that other polyesters are in use. These other polyester materials may be detected in the environment or complicate recycling streams.

### Consumer product labeling accuracy

Of the 193 polyester consumer products, 184 (95.3% [92.3% to 98.3%]) were labeled correctly, meaning the polymer identity of all components analyzed were polyesters. Seven (3.6% [1.0% to 6.2%]) were labeled entirely wrong. These included fibers identified as polypropylene from a carpet and a washcloth, cotton from two curtains, nylon-6 from a shirt, PAN from a costume hat, and a PAN/PVC blend from a dog’s stuffed toy. Two (1.0% [0% to 2.4%]) were partially wrong, including the backpack described above with a PVC component and a curtain made with a PET/cotton blend. The incorrect labels came from all consumer categories except bedding, suggesting that labeling errors are widespread across textile products. The product labels were 100% accurate for the interior or stuffing components and 94.5% [91.0% to 98.0%] accurate for the exterior polymers (5.5% [2.0% to 9.0%] were inaccurately labeled).

Many of the labels were vaguely phrased, like “contents: polyester fibers,” even though the products were made of multiple components, and some were not fibers. For example, a stuffed dog toy (‘lil Jax) labeled with this phrase was made of PAN/PVC exterior fibers, not polyesters. It is plausible that the label referred to only the stuffing. However, fibers that make up less than 5% of textile products must still be disclosed as “other fibers” on the label (https://www.ftc.gov/business-guidance/resources/threading-your-way-through-labeling-requirements-under-textile-wool-acts#section). Others have called to improve textile labeling regulations to assist the transition to a circular economy [45]. The regulations could also be improved through revising standardized lists of materials. For example, in the Generic Names for Manufactured Fibers Federal Rule (16 CFR § 303.7), the list of standardized material names contains both PLA and polyester when PLA is a type of polyester (https://www.cbp.gov/sites/default/files/assets/documents/2020-Feb/icp040_3.pdf). From chemists to manufacturers, this might cause confusion. Moreover, material labeling regulations in the U.S.A. were born in the 1930s partly from a need to protect consumers from unsafe or unsanitary filling materials, hence the commonly observed “ALL NEW MATERIALS” label. Since then, synthetic plastics have been invented and commercialized, and now, there is a great need to advance the recycling and reuse of materials. Today’s consumer and environmental protection needs and the ever-evolving redesign of polymers warrant frequent reassessments of the rules.

The accuracy of consumer product labels in this study (95.5% [92.0% to 98.0%]) was much greater than the 59% determined in a previous European study (https://www.circle-economy.com/resources/clothing-labels-accurate-or-not). The differences between the findings are likely due to methodological differences in sampling and analytical instrumentation. The European study selected end-of-life clothing of various polymer materials and fiber blends, creating a more complex, diverse, and hence challenging sample set than the current study which focused on in-use 100% polyester products. The previous study used automated sorting technology with near-infrared spectroscopy to identify polymers, a technology commonly used to sort plastics for recycling. This high-throughput method is expected to be less definitive than pyrolysis-GC/MS for identifying polymers. To our knowledge, no other studies have assessed the accuracy of textile labels for material composition. Py-GC/MS offers many advantages for future studies assessing the accuracy of product labels.

### Detection of phthalate plastic additives in polyester samples

**Fig. 6:**
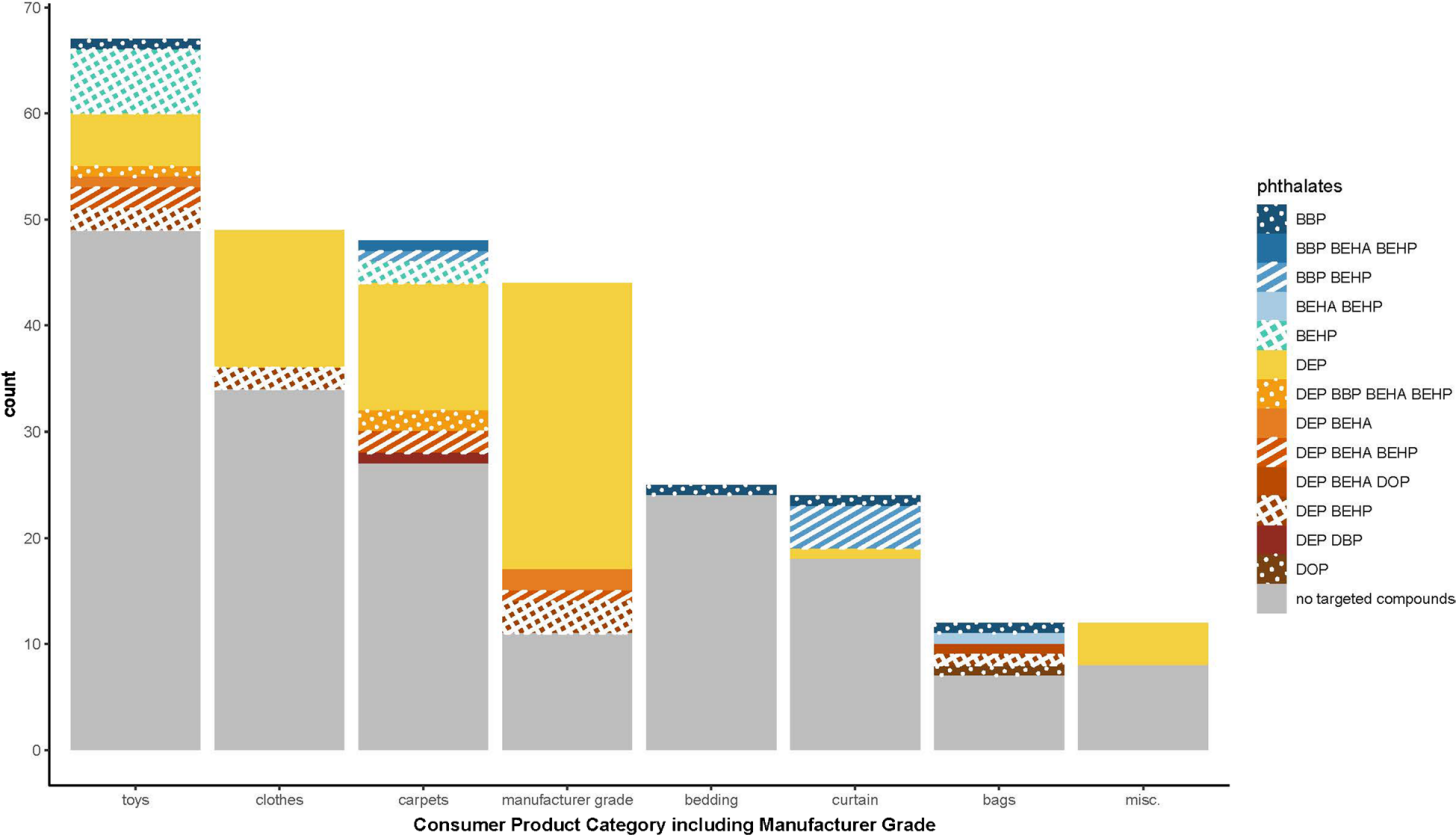
Phthalates detected in thermal desorption chromatograms of polyester-labeled manufacturer-grade and consumer product samples

DEP was the most common phthalate detected, with a detection frequency of 29.1% [23.8% to 34.4%] in the polyester samples, sometimes in the presence of other phthalates (Fig. 6). Phthalate mixtures were observed in 9.8% [6.4% to 13.3%] of the samples. Four phthalate compounds were found in two carpet fibers and one exterior component of a stuffed toy. The manufacturer-grade polyesters from the fiber library had more phthalate detections (73.1% [61.1% to 85.2%]) than the consumer goods categories, especially bedding, with the lowest detection frequency (4% [0% to 11.7%]). Most samples (63.9% [58.3% to 69.5%]) did not contain any of the targeted phthalates. The presence of additives in the manufactured-grade polyesters was unsurprising since additives are commonly added during first resin production and again during compounding or fiber production [37]. Certain phthalates, including BBP and DBP targeted here, must not exceed 0.1% by mass of children’s toys in the United States (16 CFR Part 1307 https://www.ecfr.gov/current/title-16/chapter-II/subchapter-B/part-1307). Two toys contained BBP, but this analysis was qualitative, so concentrations of phthalates in samples were not determined.

Additionally, this analysis targeted only six phthalates, meaning other classes of plastic additives or other phthalates may have been overlooked. A focus on phthalates was chosen because a preliminary screening of TD chromatograms revealed DEP in many samples. These results demonstrate the utility of the Py-GC/MS technique to perform dual analyses of the same sample, in this case, identifying the bulk polymer and characterizing plastic additives of polyester fibers. Here we assessed only the presence of phthalates, rather than quantifying their concentration, similar to most past studies using Py-GC/MS [46, 47]. To our knowledge, Py-GC/MS has been used only once to quantify plastic additive concentrations while simultaneously identifying polymers [48]. Using an external calibration curve of the targeted phthalates, and mass-labeled internal standards, would allow quantifying those plastic additives in various environmental matrices, similar to methods quantifying bulk polymer in a sample, but this was beyond the scope of the current project [25, 49, 50].

## Conclusion

In the current work, Py-GC/MS was used to identify the bulk polymer composition and plastic additive content of 285 samples labeled polyester to determine whether polyesters are primarily PET or if other aromatic polyesters are representative in commercial samples. In total, 87.4% [83.6% to 91.3%] of the samples were identified as PET, the most commonly produced polyester. Polyester blends, non-polyesters, and lone polyesters other than PET were less frequent. Most consumer products labeled “polyester” are accurately labeled, but labels neglect the type of polyester and the material composition of all components of the products. Our results suggest that Py-GC/MS can be used to determine the precise polymer identity of textiles and confirm labeling accuracy. Furthermore, this qualitative analysis demonstrates the utility of Py-GC/MS in differentiating polymers in the same class and as a tandem polymer and plastic additive identification method.

## Supplementary Information

Below is the link to the electronic supplementary material.

ESM 1 (XLSX 192 KB)

ESM 2 (PDF 1.06 MB)

## Data Availability

All pyrolysis and thermal desorption chromatograms (Masshunter) of samples considered in this study are available at https://osf.io/48ysq/?view_only=b2fdf0e4eee64da1b1e7f71aa8ddded1.

## Declarations

### NIST disclaimer

Certain commercial equipment, instruments, or materials are identified in the present study to specify adequately the experimental procedure. Such identification does not imply recommendation or endorsement by the National Institute of Standards and Technology, nor does it imply that the materials or equipment identified are necessarily the best available for the purpose.
