# Supplementary material for: Pyrolysis-GC/MS differentiates polyesters and detects additives for improved monitoring of textile labeling accuracy and plastic pollution
Source: Anal Bioanal Chem. 2025 Apr 1;417(14):3113–26. doi: 10.1007/s00216-025-05851-x (PMC12103379; doi:10.1007/s00216-025-05851-x)
Supplement: Supplementary file 2 — (PDF 1.06 MB) [file 216_2025_5851_MOESM2_ESM.pdf]

Pyrolysis-GC/MS differentiates polyesters and detects additives for improved monitoring of textile labeling accuracy and plastic pollution

Supporting Information

Josh Forakis[1], *Jennifer Lynch*[1,2]

[1]Center for Marine Debris Research, Hawaii Pacific University, 41-202 Kalanianaole Hwy #9, Waimanalo, HI 96744

[2]National Institute of Standards and Technology, 41-202 Kalanianaole Hwy #9, Waimanalo, HI 96744

Table S1. Instrument parameters for Py-GC/MS analyses

**Microfurnace Pyrolysis**	**Frontier Labs EGA/PY-3030**
Carrier gas	Helium (ultra-high purity grade)
Upper interface temperature	300 °C
Double Shot	
Thermal desorption temperature	100-200 °C
Thermal desorption program	Ramping at 20 °C min^{-1}, holding for 2 min
Pyrolysis temperature	600 °C
Pyrolysis time	0.2 min
**Gas Chromatograph**	**Agilent 8890**
Injection mode	Split; 50:1
Inlet temperature	300 °C
Oven temperature program	40 °C (2 min) → 320 °C at 20 °C min^{-1} (14 min)
Run time	30 min
Carrier gas flow and split ratio	1.000 ml min^{-1} in Helium
Column	Frontier UA5: 30 m x 250 µm x 0.25 µm
**Mass Spectrometer**	**Agilent MSD 5977**
MS source temperature	230 °C
MS Quadruple temperature	150 °C
Detection type	Scanning ion mode
Scan range	30-600 m/z
Scan rate	2.6 scans sec^{-1}
Ionization energy	70 eV

A.

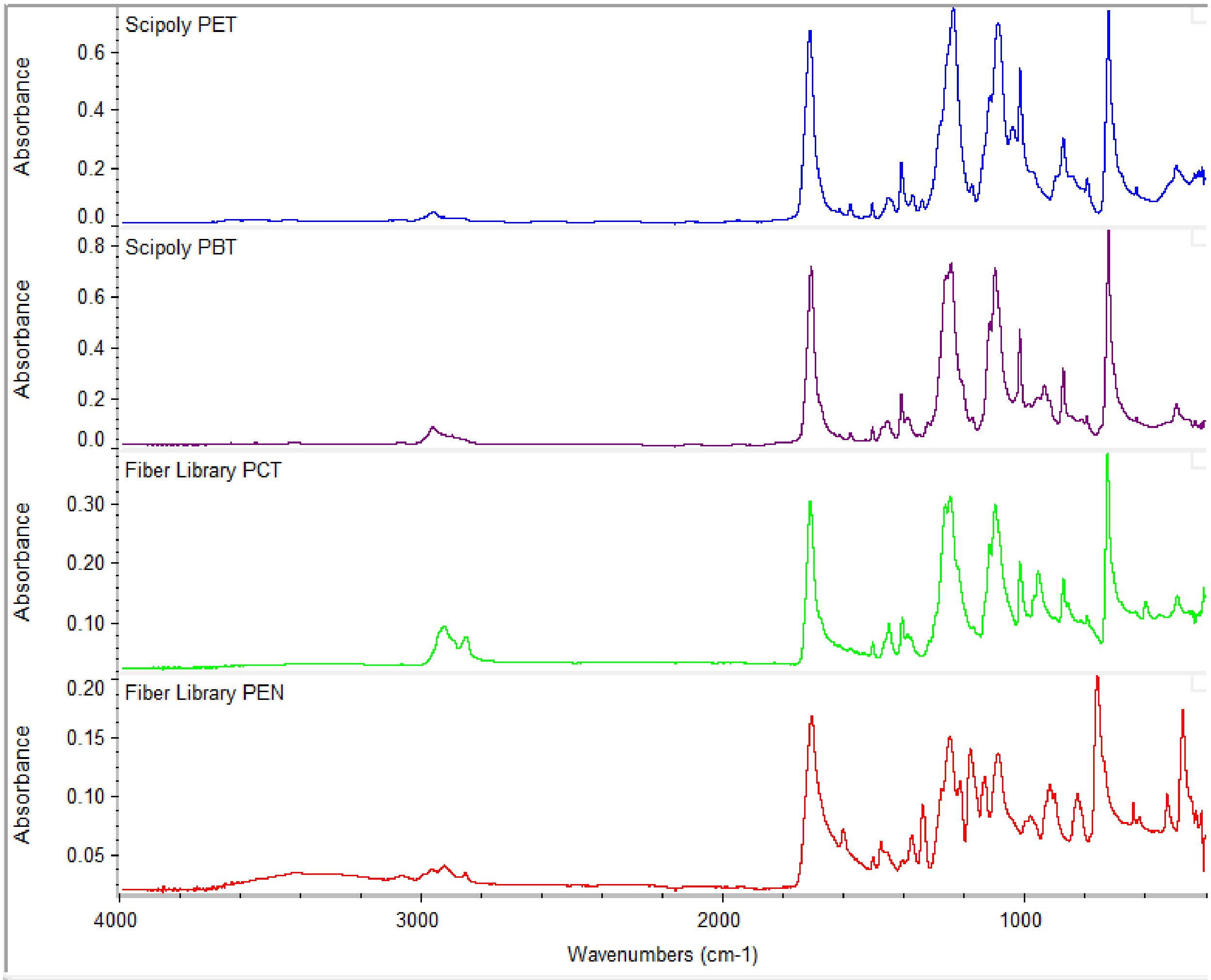

Scipoly PET
Absorbance
0.6
0.4
0.2
0.0
Scipoly PBT
Absorbance
0.8
0.6
0.4
0.2
0.0
Fiber Library PCT
Absorbance
0.30
0.20
0.10
Fiber Library PEN
Absorbance
0.20
0.15
0.10
0.05
4000
3000
2000
1000
Wavenumbers (cm-1)

B.

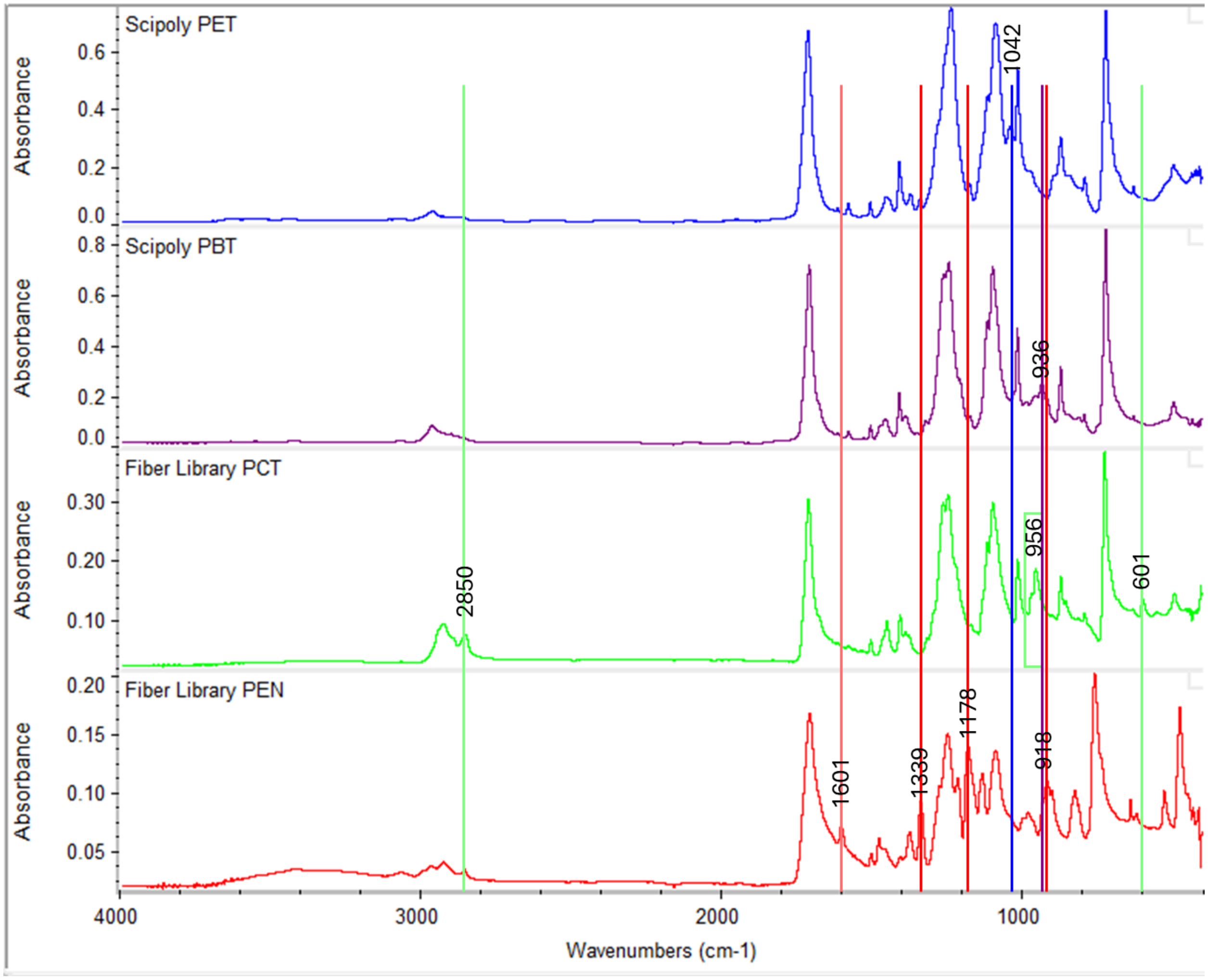
Scipoly PET
Scipoly PBT
Fiber Library PCT
Fiber Library PEN
Absorbance
Wavenumbers (cm-1)
4000
3000
2000
1000
1042
936
956
2850
601
1601
1339
1178
918

C.

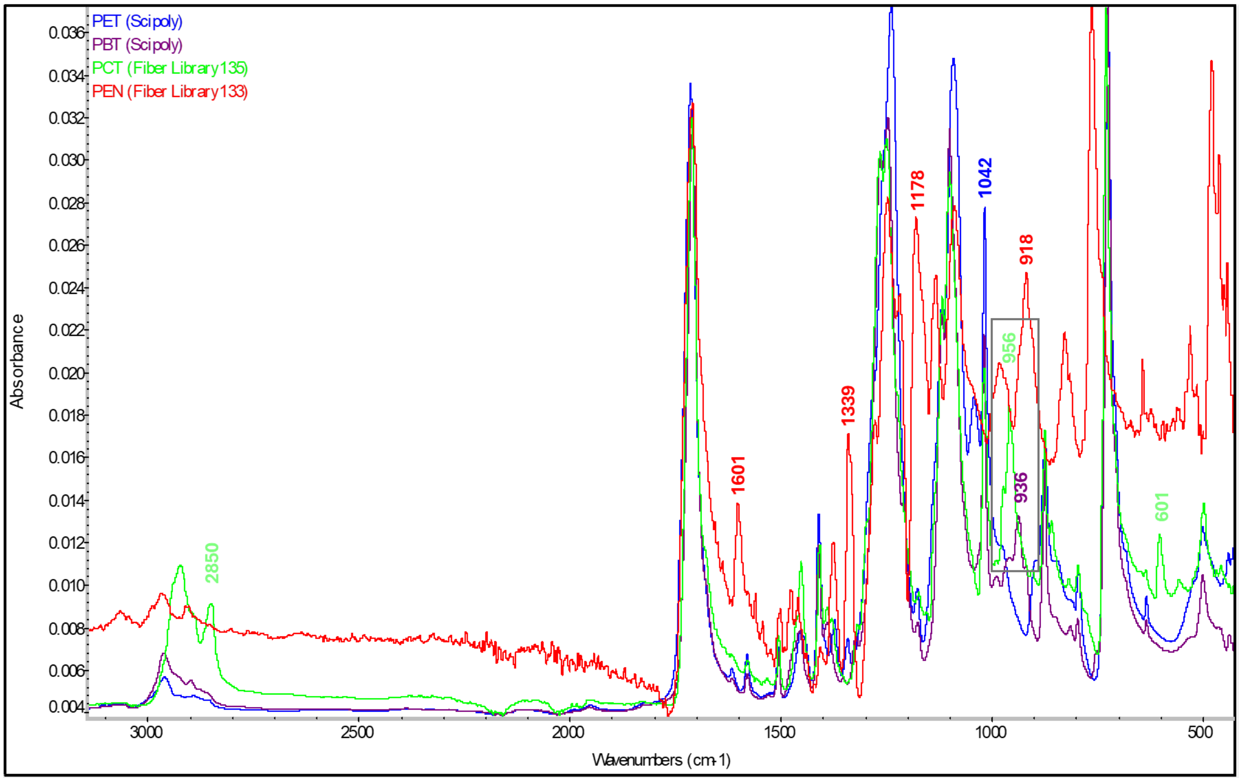

PET (Scipoly)
PBT (Scipoly)
PCT (Fiber Library135)
PEN (Fiber Library133)
Absorbance
0.036
0.034
0.032
0.030
0.028
0.026
0.024
0.022
0.020
0.018
0.016
0.014
0.012
0.010
0.008
0.006
0.004
3000
2500
2000
1500
1000
500
Wavenumbers (cm-1)
2850
1601
1339
1178
1042
918
956
936
601

D.

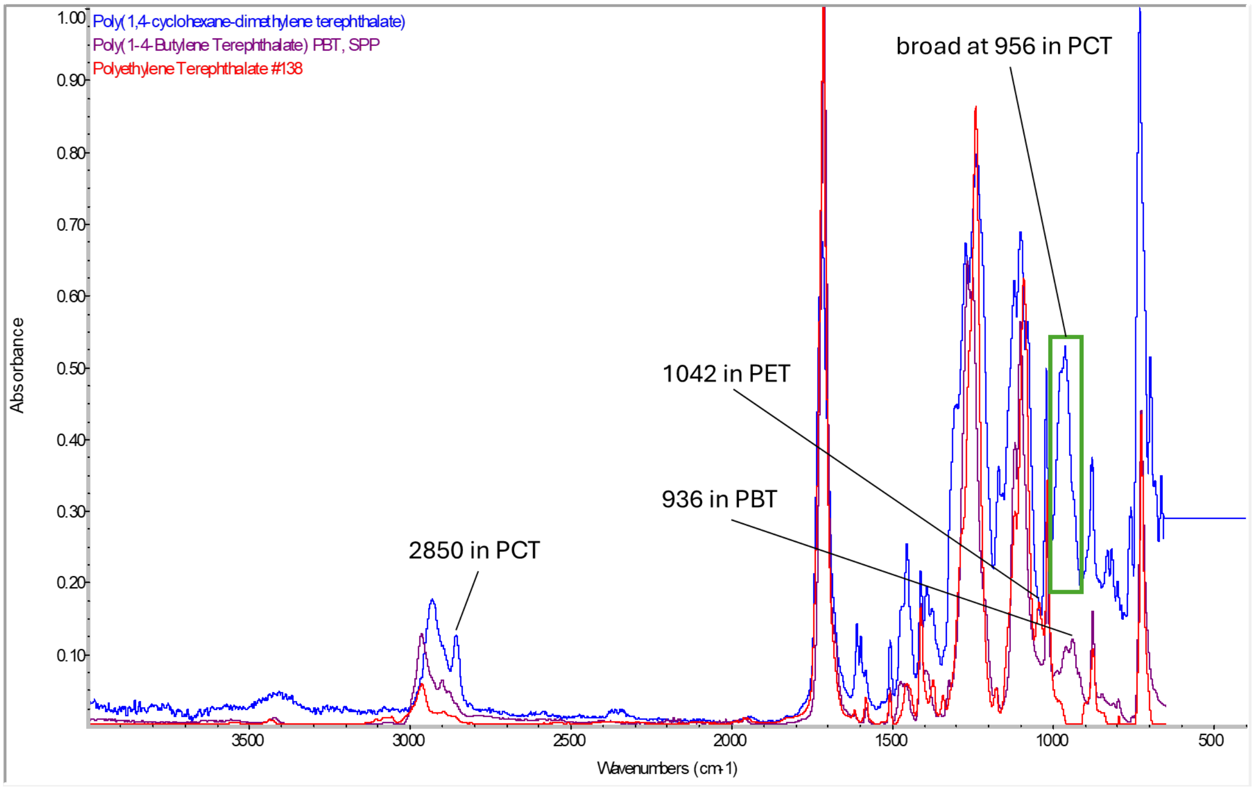

Figure S1. ATR-FTIR spectra of different polyesters. a) Spectra were collected from the four standard polyesters used in this study from 4000 - 400 cm^{-1} averaging 16 scans. Background measurements were taken, and the crystal was cleaned with 70% isopropanol between samples. b) The same spectra as “a.” c) The same spectra as “a” overlapped. d) Spectra of three polyesters from commercial spectral library (Thermo Fisher): the HR Sprouse Polymers by ATR for PCT and the HR Comprehensive Forensic FTIR Collection for PBT and PET. Graphs b-d are labeled with unique bands.

Table S2. Retention indices, molecular weight, target, and qualifier ions of polyester pyrolyzates as determined by analyzing four polyester standards by pyrolysis-GC/MS.

Polyester(s)	Pyrolyzate Name	Retention Time (min)	Retention Index	Molecular Weight (g/mol)	Target Ion (m/z)	Qualifier Ion(s) (m/z)	Relative Response of Qualifier to Target Ions (%)
PET, PCT, PBT,	benzene	2.83	688.5	78	N/A	N/A	N/A
PET, PCT	toluene	4.15	768.9	92	N/A	N/A	N/A
PET, PCT, PBT	benzoic acid	8.47	1187.7	122	N/A	N/A	N/A
PET, PCT, PBT,	biphenyl	9.68	1399.9	154	N/A	N/A	N/A
PET	Vinyl benzoate	7.81	1143.6	148	**105**	148	2
PET	Divinyl terephthalate	10.85	1586.8	218	**175**	104, 132	40, 25
PET	4-(vinyloxycarbonyl) benzoic acid	11.15	1637.8	192	**149**	65, 121	30, 20
PET	1,2-ethanediol dibenzoate	13.97	2190.2	270	**105**	227, 77	2, 25
PBT	4-(but-3-enyloxycarbonyl) benzoic acid	12.37	1859.5	220	**149**	54, 220	55, 1
PBT	dibut-3-enyl terephthalate	13.35	2057.0	274	**203**	149, 274	60, 2
PBT	1,4-butanediol dibenzoate	15.05	2443.4	298	**105**	193, 54	15, 50
PCT	bicyclo[2.2.2]octane	4.63	815.0	108	**93**	79, 77	80, 50
PCT	1,4-bis(methylene)-cyclohexane	5.10	856.5	108	**93**	79, 77	50, 70
PCT	p-xylene	5.28	872.4	106	**91**	106, 77	65, 30
PEN	naphthalene	8.28	1203.0	128	**128**	102	30
PEN	vinyl-2-naphtholate	11.56	1708.7	198	**155**	198, 127	5, 75
PEN	1,1'-binaphthalene	15.45	2544.6	254	**254**	253, 126	100, 30

Table S3. F-search results for samples that were not identified by the targeted Masshunter Quant method or the result needed confirmation.

Sample ID	Top Match	F-search ID	Hit Quality
133 Polyester Allied Signal Fibers	Pigment Blue 15:3 (C1-C20)	FLK-671	54%
135 Polyester Bayer	PCTA; [Cyclohexane dimethanol:(isophthalic acid,terephthalic acid) ; CHDM:(IPA,TPA)] (C1-C40)	FLK-284	92%
149 Polyester Grilon SA	Polyamide (C1-20)	FLY-451	94%
Fiber Library #164	Poly(butylene terephthalate) ; PBT (C1-C40)	FLG-651	78%
Elsa Backpack B1	Tube (Poly(vinyl chloride), PVC) (C1-C40)	FLE-0114	50%
Nanochemazone PCT	Polyester [(Butanediol,ethylene glycol):(adipic acid,isophthalic acid,terephthalic acid)	FLK-297	73%
Jax Stuffed Toy #3	Acrylonitrile Butadiene Rubber ; NBR 53% AN (C1-C20)	FLY-413	82%
Trafficmaster Abenaki	Polypropylene(isotactic) ; iso-PP (C1-C40)	FLG-051	75%

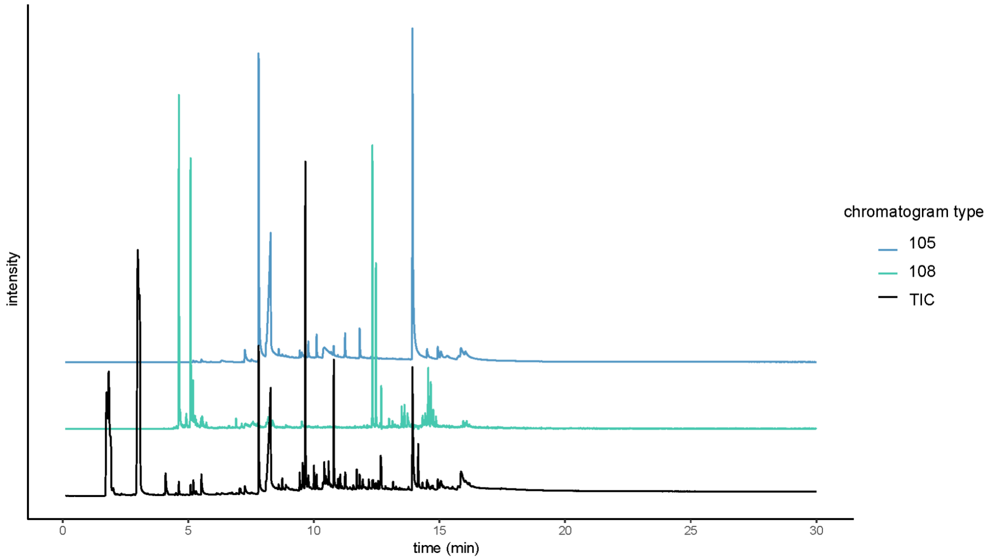

A

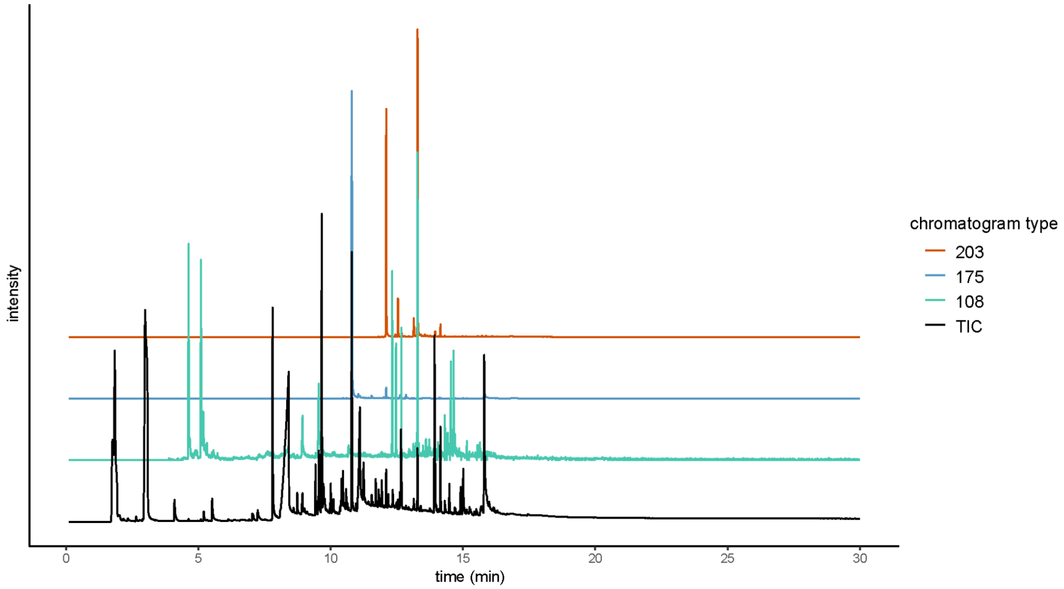

B

Figure S2. Total ion chromatograms (black) and extracted ion chromatograms of two polyester blends identified from the Microtrace Fiber Library: A) Fiber Library #147 Polyester Fiber Extrusion, B) Fiber Library #185 Polyester Wellman, Inc., identified as PET/PCT and PET/PCT/PBT, respectively. Mass ions extracted correspond to pyrolyzates unique to PBT (orange), PET (blue), and PCT (green).

Table S4. Chi square test for homogeneity of polymer ID by consumer product category. Red color denotes significance at the $p < .050$ level.

Response Dimension Label	Sample Dimension Label	LR Chisq	LR PValue	Pearson Chisq	Pearson PValue
Polymer ID by py-GC/MS	consumer product categories	73.9206	0.1068	104.961	**0.0003***

Table S5. Contingency table and comparison of polymer ID results among consumer product categories. Each consumer product category is given a letter near the bottom of the table. In the "Compare" row, a letter indicates a statistically significant difference between that column's proportion of polymer identities and the matching letter's column at the $p < 0.050$ level (capital letters) or the $p < 0.100$ level (lowercase letters). Each cell contains the count of samples, % of samples in that category, and χ^2 p-values for that cell compared to others in the same row. P-values in red are significant at the 0.050 level.

	bags	bedding	carpets	clothes	curtains	misc.	toys
sample size	12	25	48	49	24	12	67
cotton	0<br>0.0%<br>0.75032	0<br>0.0%<br>0.64601	0<br>0.0%<br>0.52449	0<br>0.0%<br>0.5202	2<br>8.3%<br>6.5e-5	0<br>0.0%<br>0.75032	0<br>0.0%<br>0.45209
PA6	0<br>0.0%<br>0.82197	0<br>0.0%<br>0.74534	0<br>0.0%<br>0.65269	1<br>2.0%<br>0.08106	0<br>0.0%<br>0.75032	0<br>0.0%<br>0.82197	0<br>0.0%<br>0.59494
PAN	0<br>0.0%<br>0.82197	0<br>0.0%<br>0.74534	0<br>0.0%<br>0.65269	1<br>2.0%<br>0.08106	0<br>0.0%<br>0.75032	0<br>0.0%<br>0.82197	0<br>0.0%<br>0.59494
PAN/PVC	0<br>0.0%<br>0.82197	0<br>0.0%<br>0.74534	0<br>0.0%<br>0.65269	0<br>0.0%<br>0.64933	0<br>0.0%<br>0.75032	0<br>0.0%<br>0.82197	1<br>1.5%<br>0.17731
PBT	0<br>0.0%<br>0.82197	0<br>0.0%<br>0.74534	1<br>2.1%<br>0.07639	0<br>0.0%<br>0.64933	0<br>0.0%<br>0.75032	0<br>0.0%<br>0.82197	0<br>0.0%<br>0.59494
PET	10<br>83.3%<br>0.82269	22<br>88.0%<br>0.93884	35<br>72.9%<br>0.22581	47<br>95.9%<br>0.6322	21<br>87.5%<br>0.91949	11<br>91.7%<br>0.93533	66<br>98.5%<br>0.43318

PET/cotton	0<br>0.0%<br>0.82197	0<br>0.0%<br>0.74534	0<br>0.0%<br>0.65269	0<br>0.0%<br>0.64933	1<br>4.2%<br>0.00474	0<br>0.0%<br>0.82197	0<br>0.0%<br>0.59494
PET/PCT	1<br>8.3%<br>0.7295	3<br>12.0%<br>0.21005	10<br>20.8%<br>0.00002	0<br>0.0%<br>0.08888	0<br>0.0%<br>0.23378	0<br>0.0%<br>0.39982	0<br>0.0%<br>0.04665
PET/PCT/PBT	0<br>0.0%<br>0.82197	0<br>0.0%<br>0.74534	1<br>2.1%<br>0.07639	0<br>0.0%<br>0.64933	0<br>0.0%<br>0.75032	0<br>0.0%<br>0.82197	0<br>0.0%<br>0.59494
PP	0<br>0.0%<br>0.75032	0<br>0.0%<br>0.64601	1<br>2.1%<br>0.3499	0<br>0.0%<br>0.5202	0<br>0.0%<br>0.65269	1<br>8.3%<br>0.00474	0<br>0.0%<br>0.45209
PVC	1<br>8.3%<br>2.45e-5	0<br>0.0%<br>0.74534	0<br>0.0%<br>0.65269	0<br>0.0<br>0.64933	0<br>0.0%<br>0.75032	0<br>0.0%<br>0.82197	0<br>0.0%<br>0.59494
Category letters	A	B	C	D	E	F	G
Compare			E	a,b,C			A,B,C,E,f

Table S6. Fisher's exact p-values comparing pairs for polymer ID results by consumer product category. Letters show all possible combinations of columns from Table S3. Red p-values indicate significance at the 0.050 level.

	cotton	PA6	PAN	PAN/ PVC	PBT	PCT	PEN	PET	PET/ cotton	PET/ PCT	PET/ PCT/ PBT	PET/ PHBa	PP	PVC
**AA**	1.0000	1.0000	1.0000	1.0000	1.0000	1.0000	1.0000	1.0000	1.0000	1.0000	1.0000	1.0000	1.0000	1.0000
**AB**	1.0000	1.0000	1.0000	1.0000	1.0000	1.0000	1.0000	1.0000	1.0000	1.0000	1.0000	1.0000	1.0000	0.3243
**BB**	1.0000	1.0000	1.0000	1.0000	1.0000	1.0000	1.0000	1.0000	1.0000	1.0000	1.0000	1.0000	1.0000	1.0000
**AC**	1.0000	1.0000	1.0000	1.0000	1.0000	1.0000	1.0000	0.7119	1.0000	0.4348	1.0000	1.0000	1.0000	0.2000
**BC**	1.0000	1.0000	1.0000	1.0000	1.0000	1.0000	1.0000	0.2325	1.0000	0.5217	1.0000	1.0000	1.0000	1.0000
**CC**	1.0000	1.0000	1.0000	1.0000	1.0000	1.0000	1.0000	1.0000	1.0000	1.0000	1.0000	1.0000	1.0000	1.0000
**AD**	1.0000	1.0000	1.0000	1.0000	1.0000	1.0000	1.0000	0.1703	1.0000	0.1967	1.0000	1.0000	1.0000	0.1967
**BD**	1.0000	1.0000	1.0000	1.0000	1.0000	1.0000	1.0000	0.3281	1.0000	0.0355	1.0000	1.0000	1.0000	1.0000
**CD**	1.0000	1.0000	1.0000	1.0000	0.4948	1.0000	1.0000	0.0018	1.0000	0.0005	0.4948	1.0000	0.4948	1.0000
**DD**	1.0000	1.0000	1.0000	1.0000	1.0000	1.0000	1.0000	1.0000	1.0000	1.0000	1.0000	1.0000	1.0000	1.0000
**AE**	0.5429	1.0000	1.0000	1.0000	1.0000	1.0000	1.0000	1.0000	1.0000	0.3333	1.0000	1.0000	1.0000	0.3333
**BE**	0.2347	1.0000	1.0000	1.0000	1.0000	1.0000	1.0000	1.0000	0.4898	0.2347	1.0000	1.0000	1.0000	1.0000
**CE**	0.1080	1.0000	1.0000	1.0000	1.0000	1.0000	1.0000	0.2320	0.3333	0.0250	1.0000	1.0000	1.0000	1.0000
**DE**	0.1050	1.0000	1.0000	1.0000	1.0000	1.0000	1.0000	0.3229	0.3288	1.0000	1.0000	1.0000	1.0000	1.0000
**EE**	1.0000	1.0000	1.0000	1.0000	1.0000	1.0000	1.0000	1.0000	1.0000	1.0000	1.0000	1.0000	1.0000	1.0000
**AF**	1.0000	1.0000	1.0000	1.0000	1.0000	1.0000	1.0000	1.0000	1.0000	1.0000	1.0000	1.0000	1.0000	1.0000
**BF**	1.0000	1.0000	1.0000	1.0000	1.0000	1.0000	1.0000	1.0000	1.0000	0.5367	1.0000	1.0000	0.3243	1.0000
**CF**	1.0000	1.0000	1.0000	1.0000	1.0000	1.0000	1.0000	0.2617	1.0000	0.1879	1.0000	1.0000	0.3627	1.0000
**DF**	1.0000	1.0000	1.0000	1.0000	1.0000	1.0000	1.0000	0.4881	1.0000	1.0000	1.0000	1.0000	0.1967	1.0000
**EF**	0.5429	1.0000	1.0000	1.0000	1.0000	1.0000	1.0000	1.0000	1.0000	1.0000	1.0000	1.0000	0.3333	1.0000
**FF**	1.0000	1.0000	1.0000	1.0000	1.0000	1.0000	1.0000	1.0000	1.0000	1.0000	1.0000	1.0000	1.0000	1.0000
**AG**	1.0000	1.0000	1.0000	1.0000	1.0000	1.0000	1.0000	0.0587	1.0000	0.1519	1.0000	1.0000	1.0000	0.1519
**BG**	1.0000	1.0000	1.0000	1.0000	1.0000	1.0000	1.0000	0.0597	1.0000	0.0183	1.0000	1.0000	1.0000	1.0000
**CG**	1.0000	1.0000	1.0000	1.0000	0.4174	1.0000	1.0000	0.0000	1.0000	0.0001	0.4174	1.0000	0.4174	1.0000

**DG**	1.0000	0.4224	0.4224	1.0000	1.0000	1.0000	1.0000	0.5726	1.0000	1.0000	1.0000	1.0000	1.0000	1.0000
**EG**	0.0674	1.0000	1.0000	1.0000	1.0000	1.0000	1.0000	0.0547	0.2637	1.0000	1.0000	1.0000	1.0000	1.0000
**FG**	1.0000	1.0000	1.0000	1.0000	1.0000	1.0000	1.0000	0.2824	1.0000	1.0000	1.0000	1.0000	0.1519	1.0000
**GG**	1.0000	1.0000	1.0000	1.0000	1.0000	1.0000	1.0000	1.0000	1.0000	1.0000	1.0000	1.0000	1.0000	1.0000

Table S7. Contingency table and comparison of polymer ID results between sample components. Each sample component is given a letter near the bottom of the table. In the "Compare" row, a letter indicates a statistically significant difference between that column's proportion of polymer identities and the matching letter's column at the $p < 0.050$ level (capital letters) or the $p < 0.100$ level (lowercase letters). Each cell contains the count of samples, % of samples in that category, and χ^2 p-values for that cell compared to others in the same row. P-values in red are significant at the 0.050 level.

	exterior	interior	stuffing
sample size	165	24	41
cotton	2<br>1.2%<br>0.63702	0<br>0.0%<br>0.64779	0<br>0.0%<br>0.55044
PA6	1<br>0.6%<br>0.73863	0<br>0.0%<br>0.74667	0<br>0.0%<br>0.67287
PAN	1<br>0.6%<br>0.73863	0<br>0.0%<br>0.74667	0<br>0.0%<br>0.67287
PAN/PVC	1<br>0.6%<br>0.73863	0<br>0.0%<br>0.74667	0<br>0.0%<br>0.67287
PET	146<br>88.5%<br>0.83745	23<br>95.8%<br>0.76324	38<br>92.7%<br>0.8563
PET/cotton	1<br>0.6%<br>0.73863	0<br>0.0%<br>0.74667	0<br>0.0%<br>0.67287
PET/PCT	10<br>6.1%<br>0.98905	1<br>4.2%<br>0.70298	3<br>7.3%<br>0.74953
PP	2<br>1.2%<br>0.63702	0<br>0.0%<br>0.64779	0<br>0.0%<br>0.55044
PVC	1<br>0.6%<br>0.73863	0<br>0.0%<br>0.74667	0<br>0.0%<br>0.67287

	A	B	C
Comparison			

Table S8. Fisher's Exact pairs for polymer ID results by sample components. Letter pairs represent all combinations of sampling components and match the columns from Table S6.

	**AA**	**AB**	**BB**	**AC**	**BC**	**CC**
cotton	1.0000	0.4598	1.0000	0.3448	.	1.0000
PA6	1.0000	0.6017	1.0000	0.5047	.	1.0000
PAN	1.0000	0.6017	1.0000	0.5047	.	1.0000
PAN/PVC	1.0000	0.6017	1.0000	0.5047	.	1.0000
PET	1.0000	0.2251	1.0000	0.4173	0.6001	1.0000
PET/cotton	1.0000	0.6017	1.0000	0.5047	.	1.0000
PET/PCT	1.0000	0.6991	1.0000	0.7711	0.6001	1.0000
PP	1.0000	0.4598	1.0000	0.3448	.	1.0000
PVC	1.0000	0.6017	1.0000	0.5047	.	1.0000
